# 异基因造血干细胞移植后BK病毒肾病1例报告及文献复习

**DOI:** 10.3760/cma.j.121090-20240810-00298

**Published:** 2025-03

**Authors:** 文丽 张, 璎玲 祖, 正华 黄, 珍 李, 瑞瑞 桂, 娟 王, 娴静 王, 会丽 王, 鑫鑫 樊, 永平 宋, 佰俊 房, 健 周

**Affiliations:** 1 郑州大学附属肿瘤医院，河南省肿瘤医院血液科，郑州 450008 Department of Hematology, The Affiliated Cancer Hospital of Zhengzhou University & Henan Cancer Hospital, Zhengzhou 450008, China; 2 郑州大学第一附属医院血液科，郑州 450052 Department of Hematology, The First Affiliated Hospital of Zhengzhou University, Zhengzhou 450052, China; 3 郑州市第三人民医院血液科，郑州 450000 Department of Hematology, The Third People's Hospital of Zhengzhou, Zhengzhou 450000, China

## Abstract

1例20岁男性患者，因“T淋巴母细胞淋巴瘤/白血病”接受无关供者HLA 9/10相合外周血造血干细胞移植，回输单个核细胞5.91×10^8^/kg，CD34^+^细胞2.88×10^6^/kg，+11 d粒细胞植入，+9 d血小板植入。移植后血肌酐反复升高，伴BK病毒（BKV）相关出血性膀胱炎和BKV血症。经肾活检、宏基因组二代测序等检查，明确诊断为BK病毒肾病（BKVN）。经调整免疫抑制剂、静脉注射免疫球蛋白、供者淋巴细胞输注等治疗，患者肾功能进行性恶化，于移植后289 d死于多器官功能衰竭。

## Abstract (en)

A 20-year-old male patient with T-lymphoblastic lymphoma/leukemia received 9/10 human leukocyte antigen-compatible unrelated peripheral blood stem cell transplantation. He was transplanted with 5.91×10^8^ mononuclear cells/kg and 2.88×10^6^ CD34^+^ cells/kg, and neutrophil engraftment was obtained at +11 days and platelet engraftment at +9 days. After transplantation, he presented with repeatedly increased serum creatinine levels, BK virus（BKV）-associated hemorrhagic cystitis, and BKV viremia. BK virus nephropathy was diagnosed based on renal biopsy and metagenomic next-generation sequencing. After adjusting the immunosuppressant, intravenous immunoglobulin, and donor lymphocyte infusion treatment, the patient's renal function deteriorated progressively, and he eventually died of multiple organ failure at +289 days.

BK病毒（BKV）是一种人类多瘤病毒，可引起超过80％的健康人无症状感染，主要潜伏于肾脏和泌尿道上皮[1]。BKV在免疫功能低下的人群例如肾移植或造血干细胞移植（HSCT）患者中可重新激活[1]。1％～10％的肾移植患者合并BK病毒肾病（BKVN），严重者可引起肾功能损伤甚至肾衰竭[1]–[2]。异基因造血干细胞移植（allo-HSCT）患者常出现BKV再激活相关出血性膀胱炎（HC），但有关BKVN的报道较少[3]–[7]。本文报告1例allo-HSCT后严重BKVN病例并进行文献复习。

## 病例资料

患者，男，20岁，2020年5月确诊为“T淋巴母细胞淋巴瘤/白血病”，VDCLP方案诱导化疗后达完全缓解（CR）。巩固化疗5个疗程，获得持续CR。2020年12月行无关供者HLA 9/10相合allo-HSCT，预处理方案：氟达拉滨（Flu）30 mg·m^−2^·d^−1^，−6 d～−2 d；白消安（Bu）3.2 mg·kg^−1^·d^−1^，−6 d、−5 d，静脉给药；美法仑（Mel）100 mg/m^2^，−4 d，静脉给药；依托泊苷100 mg·kg^−1^·d^−1^，−3 d、−2 d；抗人T细胞兔免疫球蛋白3 mg·m^−2^·d^−1^，−4 d～−1 d。减量PT/Cy（环磷酰胺15 mg·kg^−1^·d^−1^，+3 d、+4 d）联合环孢素A（CsA）和霉酚酸酯（MMF）预防移植物抗宿主病（GVHD）。回输单个核细胞5.91×10^8^/kg，CD34^+^细胞2.88×10^6^/kg。+11 d粒细胞植入，+9 d血小板植入。+30 d停用静脉CsA，改为他克莫司口服预防GVHD。+30 d发生巨细胞病毒（CMV）血症，以膦甲酸钠治疗后转阴。+36 d出现膀胱刺激征伴肉眼血尿，尿常规尿蛋白++，尿病原学培养为肺炎克雷伯菌，美罗培南治疗后转阴。但膀胱刺激征和血尿没有改善，尿BKV-DNA 8.58×10^10^拷贝数/L，诊断为HC。抗病毒和水化、碱化治疗后膀胱刺激症状缓解，但血尿持续存在。+61 d血肌酐升至183 µmol/L，予百令胶囊、谷胱甘肽等药物治疗，1个月后肌酐逐渐降至正常，+144 d肌酐再次升高至183 µmol/L，彩超示双肾积水伴双侧输尿管上段扩张，将他克莫司换为MMF预防GVHD。因肺炎克雷伯菌尿路感染口服左氧氟沙星治疗，继续百令胶囊、谷胱甘肽片等药物联合间断输注静脉注射免疫球蛋白（IVIG）治疗，但效果不佳，+260 d血肌酐达716 µmol/L，行连续肾脏替代治疗，大量输注血小板后于+268 d进行肾脏穿刺组织活检，病理示：严重肾小管损伤，上皮细胞内可见异型巨大细胞核及包涵体，肾间质弥漫水肿伴散在淋巴、单核细胞浸润（图1A、B、C）。透射电镜下可见肾小管上皮细胞微绒毛脱落，细胞核增大，核内散在分布直径30～40 nm的病毒样颗粒（图1D、E）。免疫组化显示BKV大T抗原染色肾小管上皮细胞核阳性（图1F）。同时肾组织宏基因组二代测序（mNGS）结果示：BKV总序列数122045，物种相对丰度94.54％。+280 d血BKV-DNA 1.47×10^9^拷贝数/L。综合临床症状和检验结果明确诊断为BKVN。+280 d后多次行供者淋巴细胞输注（DLI）、调整减停免疫抑制剂、床旁血液净化等治疗，患者肌酐仍持续升高，临床状况不断恶化，出现肺部感染、败血症、血压高、心功能不全、凝血功能异常、脑出血等，于+289 d死于多器官功能衰竭。

**图1:**
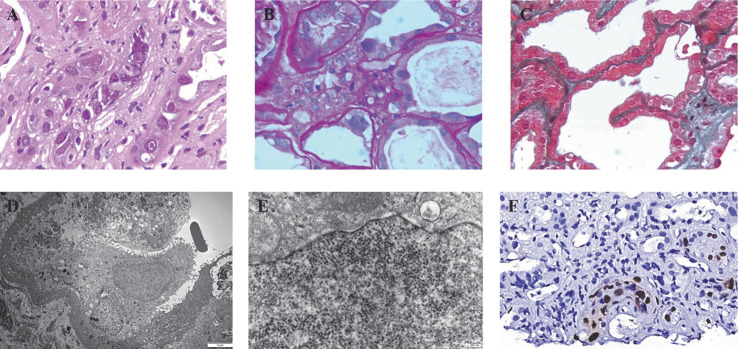
BK病毒肾病患者的肾活检组织光镜、电镜及BK病毒免疫组化染色 **A** 肾小管上皮细胞空泡、颗粒变性，可见异型巨大细胞核及包涵体（HE染色，×400）；**B** 可见蛋白管型，片状管腔扩张、细胞低平、刷状缘脱落（PAS染色，×400）；**C** 肾间质弥漫水肿伴散在淋巴、单核细胞浸润（Masson染色，×400）；**D** BK病毒感染的肾小管上皮细胞微绒毛脱落，细胞核增大；**E** 肾小管上皮细胞胞核内见病毒样颗粒；**F** BK病毒大T抗原染色肾小管上皮细胞核阳性

## 讨论及文献复习

BKVN常见于肾移植患者，allo-HSCT患者中发生率较低[3]–[7]，血液科医师对其认识不足，尤其是早期识别和诊断困难。研究报道，定量BKV血症可作为筛查肾移植患者BKVN发生的首选检测[8]，但BKV血症能否用来筛查allo-HSCT患者BKVN的发生尚需进一步研究。据文献报道[6],[9]–[11]，HSCT后BKV尿症的发生率为25％～64.8％[6],[9]–[11]，其发生的危险因素包括allo-HSCT[9]、HLA不全相合[10]、清髓性预处理[10]–[11]、CMV血症[6]、GVHD[11]、脐血干细胞移植[11]、免疫重建延迟[11]等。HSCT后BKV血症的发生率为16.9％～33.0％[6],[12]，CMV血清学阳性[12]、CMV血症[6]可能是导致其发生的高危因素。有关HSCT后发生BKVN的研究较少，且多为个案报道，但研究显示allo-HSCT后发生BKVN的患者通常合并持续性BKV血症和BKV尿症[5]–[7],[13]，提示allo-HSCT患者血肌酐明显升高合并血BKV阳性要警惕BKVN，并完善进一步检查以早期发现BKVN。肾活检是BKVN确诊的金标准。本例患者肾活检组织学发现肾小管上皮细胞内异型巨大细胞核及包涵体、电镜发现肾小管上皮细胞核内散在病毒样颗粒、BKV大T抗原免疫组化染色肾小管上皮细胞核阳性，符合BKVN肾脏病理改变。此外，肾组织mNGS结果进一步证实BKVN的诊断。但要注意的是，早期阶段肾小管上皮细胞可无病毒包涵体，电镜下也观察不到病毒样颗粒，因此，高度怀疑BKVN时建议行免疫组化及mNGS进行排查。

BKVN的治疗手段有限。目前还没有针对BKV的特异性抗病毒药物。调整或减停免疫抑制剂在某些情况下有效。研究显示西多福韦可能对肾移植患者BKV感染和BKVN有效[14]，但其在allo-HSCT后BKVN的疗效仍有争议[4]–[5],[15]。此外，研究报道免疫抑制剂来氟米特以及喹诺酮类抗生素能抑制BKV复制，IVIG可用于治疗BKV感染，但对于BKVN的疗效未尽如人意[4]–[6]。一项Ⅱ期临床试验评估了多病毒特异性T细胞疗法治疗allo-HSCT后难治性病毒感染的疗效，27例患者发生BKV相关性疾病（25例HC，2例肾炎），治疗6周后均获得部分缓解[16]。Ortí等[15]研究显示，1 × 10^6^/kg供者淋巴细胞输注能有效抑制BKV复制，他们观察到针对BKV VP1抗原的CD4^+^记忆T细胞反应性增加，但患者的临床症状仍不断加重并最终死于感染性休克。有研究评估了第三方BKV特异性细胞毒性T淋巴细胞（BKV-CTL）治疗allo-HSCT后BKV相关HC的疗效及安全性[17]，结果显示BKV-CTL输注45 d的总缓解率为81.6％（40/49），有反应的患者BKV-CTL输注后尿液中BKV载量明显下降，并且没有新发Ⅲ/Ⅳ度GVHD、植入失败和输注相关不良反应的发生。本例患者经调整免疫抑制剂、口服左氧氟沙星、IVIG及DLI等治疗效果不佳，推测该患者可能处于晚期阶段，肾功能损害严重、不可逆转。

BKVN治疗方法有限，预后不佳。早期获取病理学诊断依据并给予合理干预，避免BKVN发展至晚期，可能有助于改善预后。
